# Supplementary material for: Battling the obesity epidemic with a school-based intervention: Long-term effects of a quasi-experimental study
Source: PLoS One. 2022 Sep 27;17(9):e0272291. doi: 10.1371/journal.pone.0272291 (PMC9514666; doi:10.1371/journal.pone.0272291)
Supplement: S1 Appendix — (DOCX) [file pone.0272291.s001.docx]

**S1. Available evidence on school-based health promoting interventions**

We synthesised the evidence of systematic reviews and meta-analyses on school-based health-promoting interventions with a dietary and/or a physical activity component on effectiveness to reduce body mass index (BMI) or BMI z-score (BMIz). Studies published between January 2010 and June 2020 were reviewed in PubMed using the terms ‘BMI’, ‘Schools’, ‘Child’, and ‘Intervention’, as well as search terms related to these. In total, 218 abstracts were screened, resulting in five meta-analyses and four systematic reviews. Although the quantity of evidence is high, the quality of evidence is moderate, as a fair risk of publication bias is mentioned in four out of five meta-analyses. Three out of five meta-analyses showed that school-based interventions significantly reduce BMI, yet findings are not uniform across studies and most studies report small effect sizes. Most studies do not report effects on waist circumference, whilst this outcome measure might reflect changes in body composition better than BMI in studies involving physical activity. The majority of studies have an intervention duration ≤24 months, or rely on small study populations (figure S1); therefore, robust evidence on the trend in intervention effects with continued intervention implementation is lacking.

The meta-analyses with the highest number of included studies were used to graphically represent the average intervention duration and number of participants of school-based health-promoting interventions (figure S1).[23] 83 unique studies have been identified that reported on BMI or BMIz. The number of participants included is based on the number of participants included in the post-analysis.


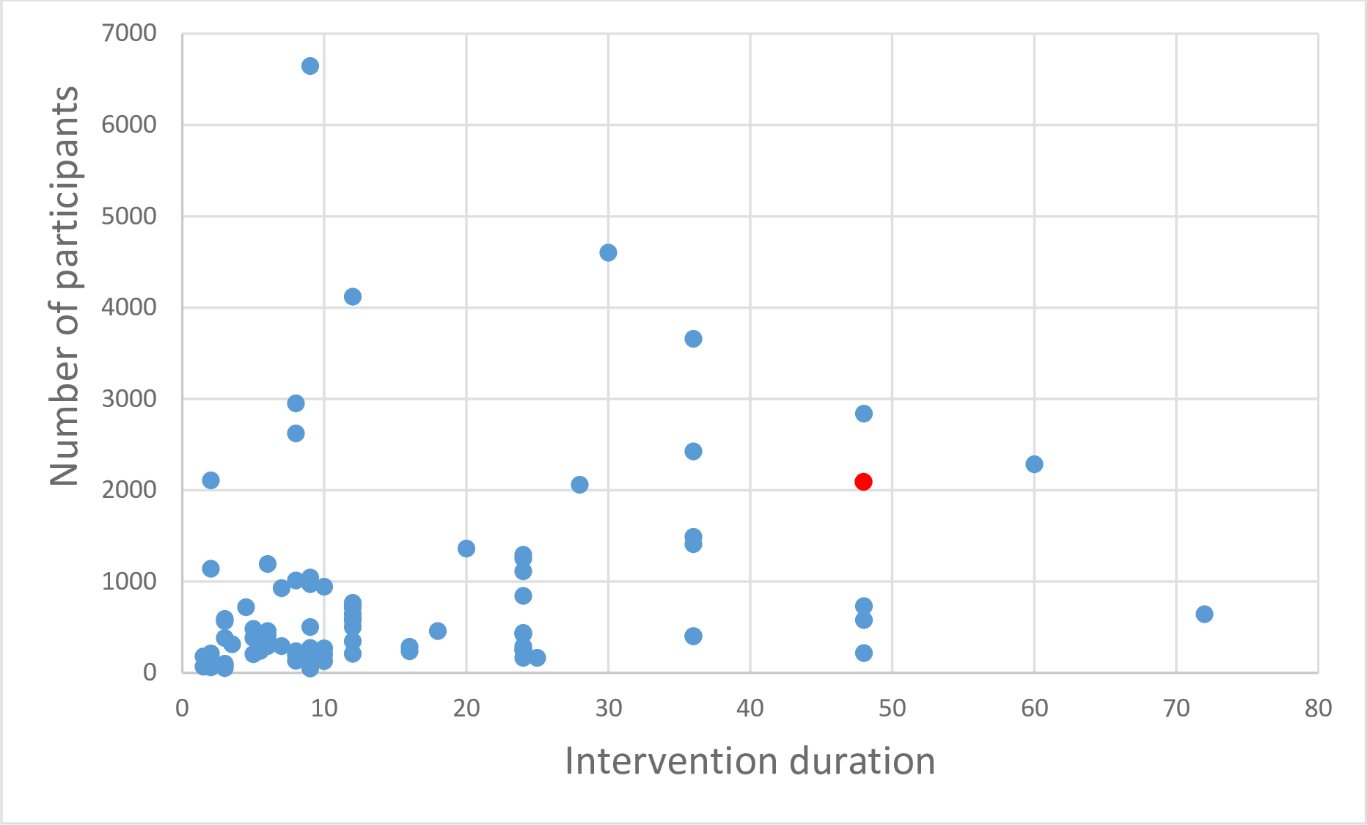


**Figure S1: Intervention duration and number of participants in school-based intervention studies, as identified by Oosterhoff et al.**

Red dot: Current study

If studies reported on the same trial, we included the study with the longest intervention duration. If intervention duration was equal, we included the most recent study. We assumed that one school year was 12 months, unless otherwise stated.

**Search terms**

| Strategy | #1 AND #2 AND #3 AND #4 |
| --- | --- |
| #1 | "Body Mass Index"[Mesh] OR "Pediatric Obesity"[Mesh] OR "Obesity"[Mesh]) OR “overweight”[Mesh] OR “BMI” [Mesh] OR “BMIz”[Mesh] OR “paediatric obesity”[Mesh] OR “adiposity”[Mesh] OR “underweight”[Mesh] OR overweight [tiab] OR body mass index [tiab] OR pediatric obesity [tiab] OR obesity [tiab] OR paediatric obesity [tiab] OR adiposity [tiab] OR underweight [tiab] OR BMI [tiab] OR BMIz [tiab] |
| #2 | “School*”[Mesh] OR “Elementary school”[Mesh] OR “Primary school”[Mesh] OR “School health services” [Mesh] OR school* [tiab] OR Elementary school [tiab] OR Primary school[tiab] OR school health services [tiab] |
| #3 | "Child*"[Mesh] OR “Youth”[Mesh] OR “Student*”[Mesh] OR child* [tiab] OR youth [tiab] OR student*[tiab ] |
| #4 | "Intervention*"[Mesh] OR Intervention* [tiab] |

**Inclusion criteria**

| **Study design** | Systematic review or meta-analysis |
| --- | --- |
| **Language** | Dutch or English |
| **Publication date** | January 2010 and June 2020 |
| **Outcome measure** | BMI, BMIz, overweight, underweight, obesity or adiposity. |
| **Population of included studies** | Children aged 4-12 years. |
| **Design of included studies** | Longitudinal studies with a pretest posttest design and a control group. |
| **Interventions of included studies** | Health-promoting interventions designed to promote healthy diet and/or physical activity, or prevent obesity, conducted in a school-based setting aimed at the entire school population. |

**Exclusion criteria**
- Studies in which solely boys or girls were included.
- If several age groups were included, reviews/meta-analyses were excluded if results were not presented separately for children aged 4-12 years old.
- Reviews or meta-analyses that exclusively included overweight or obese children.
- Reviews or meta-analyses which included studies that also focused on interventions outside the school setting.
- Reviews and meta-analyses solely focusing on moderating factors.

**Flowchart S1.**





**Table S1: Characteristics of included studies**^a^

| **Authors** | **Year of publication** | **Type of study** | **Population** | **N included** | **Intervention and intervention duration** | **Bias** | **Pooled estimate** | **Conclusion** |
| --- | --- | --- | --- | --- | --- | --- | --- | --- |
| M. Oosterhoff  M. Joore  I. Ferreira^b^[23] | 2016 | Meta-analysis | Children aged 4-12 years old | Studies: 83 trials in 88 papers. (85 trials were included in the paper in total.)  Number of participants (in all 85 trials included): 72934 | Interventions implemented in the school setting: A lifestyle intervention was defined as any including changes towards healthier eating, physical activity and inactivity levels and/or education for healthier dietary and activity behaviours. These interventions could vary in content and mode of delivery.  Mean intervention duration (for all 85 trials included): 16.5 months (range from 1.5–96 months) | The pooled effect sizes seemed not to have been threatened by publication bias. There was large heterogeneity of the effects of school-based interventions on pupils’ changes in BMI and BMIz. Aspects of methodological quality did not explain any significant portion of the heterogeneity. | SMD for BMI and BMIz multivariate analysis:  -0.072 (95%CI: -0.106 to -0.038), p<0.001 | Overall, the authors found that school-based lifestyle interventions induced favourable changes in BMI and BMIz. |
| R. Martin,  EM. Murtagh[33] | 2017 | Systematic review | Children aged 5-12 years old | Studies: 3  Number of participants: 6980 | All included interventions were required to involve the teaching of academic lessons with the integration of PA. The classroom-based PA intervention was the sole intervention of all included studies.  Mean intervention duration: 20 months^d^ (range from 1-3 years) | There is a limited number of studies included in the review particularly for BMI and consequently conclusions are drawn based on a small number of articles. Many of the included studies were assessed to be of high risk of bias in many areas according to the Cochrane Collaboration assessment tool or did not include sufficient information to make firm judgements about bias. Therefore, the results reported must be interpreted with caution since less rigorous studies may be biased toward overestimating or underestimating true intervention effects. Publication bias was not assessed but might have been a problem. | N/A | All three studies which examined intervention effects on students’ BMI reported some positive results with small effect sizes. |
| A. Verrotti  L. Penta  L. Zenzeri  S. Agostinelli  P. De Feo[34] | 2014 | Systematic review | Children until 12 years of age | Studies: 35  Number of participants: unknown | Any intervention delivered in a school setting (all studies focused on nutrition, physical activity and/or sedentary behaviour).  Mean intervention duration: 21.5 months^d^ (range from 4 months-6 years) | The studies found were heterogeneous in terms of design, interventions and outcomes. | N/A | The findings are inconsistent, but overall suggest that combined diet and physical activity interventions (especially in collaboration with health professionals) with educational programmes and family involvement may help to prevent children becoming overweight in the long term, because all these factors can modify an ‘obesogenic’ lifestyle. |
| H. Mei,  Y. Xiong,  S. Xie,  S. Guo,  Y. Li,  B. Guo  J. Zhang[35] | 2016 | Meta-analysis | Children aged 6-12 years old | Studies: 18  Number of participants: 22381 | Interventions conducted in the school setting with a PA intervention duration > 12 months. Five studies (27.8%) only included PA intervention and the other 13 (72.2%) involved both PA and diet components.  Mean intervention duration: unknown. 9 studies had a duration of 12-24 months and 9 studies had a duration of > 24 months. (range from 12-72 months) | The heterogeneity was high across the studies and the asymmetric funnel plot indicated that studies included in the analysis may have publication bias. The low quality of the included studies was also of concern in the current analysis. | SMD for BMI: -2.23 kg/m^2^, (90 % CI: -2.92 to -1.56), p<0.05. | The authors found an overall significant impact of long-term PA intervention on reducing BMI gain among primary school children. In their study, the PA&N intervention durations that lasted for at least one year had a positive impact on children’s BMI. Weekly PA intervention for both ≤100 min and >100 min reduced children’s BMI increment significantly, while the reduction of BMI increment was higher in the group with intervention ≤100 min. |
| S. Sobol-Goldberg  J. Rabinowitz  R. Gross[8] | 2013 | Meta-analysis | Children aged > 5 years | Studies: 18  Number of participants: 30780 | School-based obesity prevention programmes: The interventions included in the studies were designed to reduce body mass by altering lifestyle. This includes changing eating habits by increasing intake of healthy foods and decreasing consumption of unhealthy foods; and by changing patterns of activity to more physical and less sedentary.  Mean intervention duration (for all 32 studies included): unknown (range from less than 1 year – 4 years) | Of the 32 studies included, only two (6%) studies had allocation concealment, two (6%) had participant or provider blinding, five (15%) had blinding of data collectors, and seven (21%) had loss to follow-up under 20%.  There was support for a lack of publication bias and the study included largely heterogeneous studies. | SMD for BMI: -0.104 kg/m^2^ (95% CI: -0.195 to -0.01);p< 0.05 | The 18 studies focusing exclusively on children reported a significant BMI decline.  Authors found that long-term interventions – lasting 1-4 years – were more effective than shorter ones (this conclusion was drawn for all studies, including studies concerning adolescents). |
| S. Verjans-Janssen  I. van de Kolk  DHH. van Kann  SPJ Kremers  SMPL Gerards[36]^c^ | 2018 | Systematic review³ | Children aged 4-12 years old | Number of studies: 18  Number of participants: 34361 | School-based interventions targeting physical activity behaviour, sedentary behaviour and/or nutrition behaviour that directly involved parents.  Mean intervention duration: 20 months^d^ (range from 12 weeks - 3.5 years) | Authors mention a risk of publication bias. Another limitation may be the inclusion of studies with a weak methodology as often information was missing. | N/A | Eleven of the 18 studies measuring intervention effects on BMI or BMIz found favourable results. Of these, seven studies were positively effective on BMI and BMIz and four studies found mixed results. Of the studies with favourable results, effect sizes for BMI or BMIz were mainly small (ES -0.04 to -0.27). Two studies had a moderate effect size (ES -0.34 and -0.48) and one study found a large effect on BMI (ES -0.79). Six studies reported their intervention to be ineffective regarding BMI and BMIz, although three of these showed a positive trend (ES -0.10 and -0.01, respectively). One study found negative results.  It seems that interventions of longer duration (at least one year) were more likely to lead to favourable results regarding weight status |
| LS. Hung  DK. Tidwell  ME. Hall  ML. Lee  CA. Briley  BP. Hunt[22] | 2015 | Meta-analysis | Children aged > 6 years. Mean age < 10 years. | Number of studies: 16  Number of participants: 14229 | Programmes were school-based with a dietary and/or physical activity component.  Mean intervention duration: unknown (6 studies lasted < 1 year and 10 studies lasted > 1 year) | An asymmetrical funnel plot indicated publication bias. Smaller studies were lacking. This meta-analysis was heterogeneous.  The conclusions that can be drawn are limited by the robustness of the studies used for the analyses. | SMD^e^ for BMI or skinfold thickness of children <10 years old: -0.036 (95% CI: 0.095 to -0.167); p>0.05. | Considering the small effect size that was observed, the authors concluded that school-based interventions have not been effective in combatting childhood obesity. However, school-based RCTs that focused only on physical activity or nutrition appeared to produce promising results for impacting childhood obesity. |
| VL. Errisuriz  NM. Golaszewski K. Born JB. Bartholomew[37] | 2018 | Systematic review | Students from PE classes in elementary (or primary) schools aged 6-11 years. | Number of studies: 10   Number of participants: 7341^f^ | Interventions with a deliberate attempt to change usual teaching practice in PE with the intention of either increasing PA or fitness. Interventions had to be delivered by school-employed, specialist PE teachers.  Mean intervention duration^d^: 25 months (range from 26 weeks – 156 weeks) | Of the ten studies, four were classified as low quality, five as medium quality, and one as high quality.  The heterogeneous nature and lack of appropriate evaluation of the intervention designs reviewed make it difficult to determine which programmes or components are most effective. | N/A | The majority of studies demonstrate significant body composition differences between intervention and control groups using BMI. Considering that maturation impacts BMI, it is not surprising that all studies showed increased scores. Six of ten studies measuring body fat percentage or BMI reported an increase among both intervention and control group students. It is notable that most PE interventions reduced this increase. Studies using skinfold thickness and body fat percentage had consistent findings, indicating that PE interventions had a minimal impact on body composition. |
| G. Sbruzzi. B. Eibel SM. Barbiero RO. Petkowicz RA. Ribeiro CC. Cesa et al.[38] | 2013 | Meta-analysis | Children aged 6-12 years old | BMI: 15, BMI z-score: 6 WC: 5  Number of participants BMI: 18423 BMI z-score: 5892 WC: 4523 | Interventions in a school-based programme with the aim of addressing overweight and obesity. The programmes were considered for inclusion if there was any deliberate approach to increase physical activity, decrease participation in sedentary activities, improve dietary behaviours, decrease intake of dietary fat and sugar, or a combination of the above approaches.  Mean intervention duration (for studies involving BMI)^d^: 20.5 months (range from 6 months – 72 months) | The quality of the evidence for waist circumference, BMI z-score and BMI was considered very low (partly based on potential for publication bias). There was high heterogeneity between studies. | SMD BMI: -0.07 kg/m^2^ (95% CI -0.19, 0.05); p = 0.26   SMD BMIz: -0.04 kg/m^2^ (95% CI -0.13 to 0.05);   p =0.37  SMD WC: -0.98 cm (95% CI -2.20 to 0.24);  p = 0.11 | In conclusion, educational interventions are not effective in preventing childhood obesity and its consequences.  In sensitivity analyses considering duration of intervention, the authors observed that educational interventions performed for longer than 12 months were associated with reduction in BMI, which was not observed in studies that test interventions for less than 12 months. |

^a^ Some studies included a broader population. The details in this table concern solely our target group unless otherwise stated. This does not apply to ‘bias’ since information was often not provided for different subgroups separately.
^b^ The meta-analysis included three RCTs which targeted children with only overweight or obesity. We decided on inclusion of this meta-analysis, as these three studies will most likely affect the conclusions of this meta-analysis minimally, as a total of 83 studies are included.

^c^ The systematic review included two studies without a control group. We decided to include this systematic review, as the authors already acknowledged the limited use of these studies in their own conclusions.
^d^ Intervention duration was calculated by the researchers of the present study since it was not mentioned in the article. We assumed that one school year was 12 months.

^e^As some meta-analyses labelled a negative SMD as beneficial for the intervention group, whilst other meta-analyses labelled a positive SMD as beneficial for the intervention group, we converted all SMDs in such a way that a negative SMD is considered beneficial for the intervention group.

^f^ For one study, the N was not reported.

Abbreviations: BMI = body mass index, CI = confidence I=interval, PA = physical activity, N/A = not applicable, SMD = standardised mean difference, WC = waist circumference.

**References**

8. Sobol-Goldberg S, Rabinowitz J, Gross R. School-based obesity prevention programs: a meta-analysis of randomized controlled trials. Obesity (Silver Spring, Md). 2013;21(12):2422-8. Epub 2013/06/26. doi: 10.1002/oby.20515. PubMed PMID: 23794226.

22. Hung LS, Tidwell DK, Hall ME, Lee ML, Briley CA, Hunt BP. A meta-analysis of school-based obesity prevention programs demonstrates limited efficacy of decreasing childhood obesity. Nutrition research (New York, NY). 2015;35(3):229-40. Epub 2015/02/07. doi: 10.1016/j.nutres.2015.01.002. PubMed PMID: 25656407.

23. Oosterhoff M, Joore M, Ferreira I. The effects of school‐based lifestyle interventions on body mass index and blood pressure: a multivariate multilevel meta‐analysis of randomized controlled trials. Obes Rev. 2016;17(11):1131-53.

33. Martin R, Murtagh EM. Effect of Active Lessons on Physical Activity, Academic, and Health Outcomes: A Systematic Review. Research quarterly for exercise and sport. 2017;88(2):149-68. Epub 2017/03/23. doi: 10.1080/02701367.2017.1294244. PubMed PMID: 28328311.

34. Verrotti A, Penta L, Zenzeri L, Agostinelli S, De Feo P. Childhood obesity: prevention and strategies of intervention. A systematic review of school-based interventions in primary schools. J Endocrinol Invest. 2014;37(12):1155-64. Epub 2014/09/10. doi: 10.1007/s40618-014-0153-y. PubMed PMID: 25200996.

35. Mei H, Xiong Y, Xie S, Guo S, Li Y, Guo B, et al. The impact of long-term school-based physical activity interventions on body mass index of primary school children - a meta-analysis of randomized controlled trials. BMC Public Health. 2016;16:205. Epub 2016/03/05. doi: 10.1186/s12889-016-2829-z. PubMed PMID: 26931236; PubMed Central PMCID: PMCPMC4774105.

36. Verjans-Janssen SR, van de Kolk I, Van Kann DH, Kremers SP, Gerards SM. Effectiveness of school-based physical activity and nutrition interventions with direct parental involvement on children’s BMI and energy balance-related behaviors–A systematic review. PLoS One. 2018;13(9):e0204560.

37. Errisuriz VL, Golaszewski NM, Born K, Bartholomew JB. Systematic Review of Physical Education-Based Physical Activity Interventions Among Elementary School Children. The journal of primary prevention. 2018;39(3):303-27. Epub 2018/05/01. doi: 10.1007/s10935-018-0507-x. PubMed PMID: 29705883.

38. Sbruzzi G, Eibel B, Barbiero SM, Petkowicz RO, Ribeiro RA, Cesa CC, et al. Educational interventions in childhood obesity: a systematic review with meta-analysis of randomized clinical trials. Prev Med. 2013;56(5):254-64. Epub 2013/03/05. doi: 10.1016/j.ypmed.2013.02.024. PubMed PMID: 23454596.
