# Supplementary material for: Battling the obesity epidemic with a school-based intervention: Long-term effects of a quasi-experimental study
Source: PLoS One. 2022 Sep 27;17(9):e0272291. doi: 10.1371/journal.pone.0272291 (PMC9514666; doi:10.1371/journal.pone.0272291)
Supplement: S2 Appendix — (DOCX) [file pone.0272291.s002.docx]

**S2. Data collection, outcomes and covariates**

**Data collection**

All measurements were conducted in children from study year two to eight, unless stated otherwise in table S2. For children in study year one, data was only collected via the parental questionnaire. All child questionnaires were filled out by hand during class hours. The anthropometric measurements (weight, height, WC) were performed twice; a third measurement was conducted if the difference between the two measurements exceeded a pre-set limit (weight ≥ 0.2 kg, height ≥ 0.5 cm, WC ≥ 1.0 cm). Children were measured while wearing light sports clothes and no shoes. WC was measured using a measuring tape, in compliance with the WHO assessment protocol. Due to measurement errors in 2015, data of WC in this year is lacking in four schools. PA was measured by the ActiGraph GT3X+ accelerometer (30 Hz, 10 s epoch), attached to the hip with an elastic band.

**Table S2: School groups* in which annual measurements were conducted**

|  | **Anthropometry** | **Accelerometry** | **Parent questionnaire** | **Child questionnaire** | **Child lunch questionnaire** |
| --- | --- | --- | --- | --- | --- |
| **T0** | 2-8  Waist and hip circumference data are lacking in four schools due to measurement errors | 2-8 | 1-8 | 4-8 | 3-8 |
| **T1** | 2-8 | 2-8 | 1-8 | 4-8 | 3-8 |
| **T2** | 2-8 | 2-8 | 1-8 | 4-8 | 3-8 |
| **T3** | BMI: 2-8  Waist circumference: 2-8  Hip circumference: 2 and 8 | 2 and 8 | 1-8 | 4-8 | 3-8 |
| **T4** | 3-8 | 3-8 | 2-8 | 4-8 | 3-8 |

* Children in Dutch schools are divided over 8 groups, whereby children in group 1 and 2 are aged 4-6 years old, which correspond to kindergarten, and children in Dutch groups 3-8 are aged 6-12 years which corresponds to the International recognized grade 1-6.

**Outcomes**

*Children’s body composition*

Children’s BMI was calculated using height and weight. Weight categories were based on internationally recognised cut-off points.[39, 40]

*Children’s PA behaviours*

Children wore the monitor continuously for seven days starting on Monday, except during sleeping hours or water activities. Minimal wear time was defined as 480 min per day between 6am and 11pm for a minimum of three valid weekdays and one valid weekend day, following Choi’s classification criteria.[41] The first day of each measurement period was excluded to prevent reactivity and/or over-reactivity. Data was processed using ActiLife version 16.3.3 (ActiGraph, Pensacola, FL, USA). PA was classified into percentage of the day spent in sedentary behaviour (SB), light PA (LPA) and moderate to vigorous PA (MVPA) using internationally recognised cut-off points.[42]

*Children’s dietary behaviours*

Water consumption during school hours was derived from the child questionnaire ranging from never (0) to every day (3). As a healthy diet is best represented by a combination of dietary habits, the composite scores of healthy dietary behaviours, unhealthy dietary behaviours and lunch intake have been generated from both parent and child data, ranging from never (0) to every day (7). The composite scores of ‘healthy and unhealthy dietary behaviours’ are derived from the online parent questionnaire. The items from this questionnaire originate from the Local and National Youth Health Monitor and included a short food-frequency questionnaire over the previous week.[43] The ‘healthy dietary behaviours’ outcome was composed by averaging the weekly consumption (ranging from 0-7 days/week) of breakfast consumption, intake of fruits, cold and warm vegetable intake, and water intake throughout the day. The ‘unhealthy dietary behaviours’ outcome was composed by averaging the weekly consumption (ranging from 0-7 days/week) of sugar-sweetened beverages and four different snack types (chocolate, salted snacks, cookies and soft ice creams).

Lunch intake at school was derived from the questionnaires for children and was registered using recall data from children immediately after lunch. The items bread and cereals were combined into the food type ‘grains’, and milk/yoghurt and cheese were combined into the food type ‘dairy’. The items were summarised into six dichotomous (yes/no) food types: fruits, vegetables, grains, dairy, water and butter. To give an indication of the nutritional value of children’s lunches, the six food types were summed to create a dichotomous variable reflecting consumption of at least two food types during lunch.

*Covariates*

Birthdate, sex, and study year of children were gathered via the school system and checked for correctness with data from the regional Youth Health Department. Ethnicity and socioeconomic status were collected via the parental questionnaire. Ethnicity was divided into Western and non-Western, as defined by having minimally one parent born in a non-Western country.[44] Socioeconomic status was calculated using a standardised score of maternal education level, paternal education level and household income adjusted for household size.[45] Mean scores were categorised into low, middle and high, based on tertiles. To control for the influence of weather on PA measurements, daily regional weather conditions of the measurement week were obtained from the Royal Dutch Meteorological Institute (temperature, sun exposure and precipitation).

**References**

39. Cole TJ, Bellizzi MC, Flegal KM, Dietz WH. Establishing a standard definition for child overweight and obesity worldwide: international survey. BMJ. 2000;320(7244):1240.

40. Cole TJ, Flegal KM, Nicholls D, Jackson AA. Body mass index cut offs to define thinness in children and adolescents: international survey. Bmj. 2007;335(7612):194. Epub 2007/06/27. doi: 10.1136/bmj.39238.399444.55. PubMed PMID: 17591624; PubMed Central PMCID: PMCPMC1934447.

41. Choi L, Liu Z, Matthews CE, Buchowski MS. Validation of accelerometer wear and nonwear time classification algorithm. Med Sci Sports Exerc. 2011;43(2):357.

42. Evenson KR, Catellier DJ, Gill K, Ondrak KS, McMurray RG. Calibration of two objective measures of physical activity for children. J Sports Sci. 2008;26(14):1557-65.

43. Lokale en Nationale Monitor Jeugdgezondheid [Local and National Youth Health Monitor]. Standaardvraagstelling Voeding [Standard Questionnaire Nutrition] [cited 2015 16 March]. Available from: <https://www.monitorgezondheid.nl/jeugdindicatoren.aspx>

44. Keij I. Hoe doet het CBS dat nou? Standaarddefinitie allochtonen [How does Statistics Netherlands do this? Standard definition of emigrants]. 2000.

45. Shavers VL. Measurement of socioeconomic status in health disparities research. J Natl Med Assoc. 2007;99(9):1013.
