# Supplementary material for: Battling the obesity epidemic with a school-based intervention: Long-term effects of a quasi-experimental study
Source: PLoS One. 2022 Sep 27;17(9):e0272291. doi: 10.1371/journal.pone.0272291 (PMC9514666; doi:10.1371/journal.pone.0272291)
Supplement: S3 Appendix — (DOCX) [file pone.0272291.s003.docx]

**S3. Changes in weight category according to BMI categories after one and four years of exposure**

**Table S3a: Changes in weight category according to BMI after one year of exposure, in %**

|  | **Total population (n=1163)** | **Full HPSF (n=369)** | **Partial HPSF (n=352)** | **Control (n=442)** |
| --- | --- | --- | --- | --- |
| Maintenance of weight category | 89·0 | 89·4 | 88·1 | 89·4 |
| Development of underweight | 3·4 | 3·3 | 3·7 | 3·4 |
| Development of overweight or obesity | 3·1 | 3·5 | 2·8 | 2·7 |
| Normal weight developed from underweight | 1·3 | 1·1 | 0·9 | 2·0 |
| Normal weight developed from overweight or obesity | 3·2 | 2·7 | 4·5 | 2·5 |

Bold value = significant (≤0·05) difference between conditions

Abbreviations: HPSF, Healthy Primary School of the Future.

**Table S3b: Changes in weight category according to BMI after four years of exposure, in %**

|  | **Total population (n=1163)** | **Full HPSF (n=369)** | **Partial HPSF (n=352)** | **Control (n=442)** |
| --- | --- | --- | --- | --- |
| Maintenance of weight category | 76·4 | 76·1 | 75·7 | 76·3 |
| Development of underweight | 5·1 | 5·1 | 4·7 | 5·3 |
| Development of overweight or obesity | 10·4 | 10·3 | 9·5 | 11·2 |
| Normal weight developed from underweight | 1·4 | 1·7 | 0·7 | 3·3 |
| Normal weight developed from overweight or obesity | 6·7 | 6·8 | 9·5 | 3·9 |

Bold value = significant (≤0·05) difference between conditions

Abbreviations: HPSF, Healthy Primary School of the Future.
