# Supplementary material for: Battling the obesity epidemic with a school-based intervention: Long-term effects of a quasi-experimental study
Source: PLoS One. 2022 Sep 27;17(9):e0272291. doi: 10.1371/journal.pone.0272291 (PMC9514666; doi:10.1371/journal.pone.0272291)
Supplement: S4 Appendix — (DOCX) [file pone.0272291.s004.docx]

**S4. Estimated intervention trend on BMIz stratified by sex**

**Table S4: Estimated intervention trend on BMIz stratified by sex**

|  | | **Full HPSF vs control** | | | **Partial HPSF vs control** | | | |
| --- | --- | --- | --- | --- | --- | --- | --- | --- |
|  |  | **B (95% CI)** | ***p*** | **ES** | **B (95% CI)** | ***p*** | **ES** |  |
| **BMIz for boys**  **(n=)** | **E1** | -0·055 (-0·091 to -0·018) | **0·004** | -0·06 | -0·031 (-0·068 to 0·005) | 0·09 | -0·03 |  |
|  | **E2** | -0·109 (-0·183 to -0·035) | **0·004** | -0·12 | -0·063 (-0·135 to 0·010) | 0·09 | -0·07 |  |
|  | **E3** | -0·164 (-0·274 to -0·053) | **0·004** | -0·18 | -0·094 (-0·203 to 0·015) | 0·09 | -0·10 |  |
|  | **E4** | -0·218 (-0·366 to -0·070) | **0·004** | -0·24 | -0·125 (-0·270 to 0·020) | 0·09 | -0·14 |  |
| **BMIz for girls**  **(n=2094)** | **E1** | -0·026 (-0·056 to 0·004) | 0·09 | -0·03 | -0·047 (-0·078 to -0·017) | **0·002** | -0·05 |  |
|  | **E2** | -0·052 (-0·112 to 0·008) | 0·09 | -0·05 | -0·095 (-0·155 to -0·034) | **0·002** | -0·10 |  |
|  | **E3** | -0·078 (-0·168 to 0·012) | 0·09 | -0·08 | -0·142 (-0·233 to -0·051) | **0·002** | -0·15 |  |
|  | **E4** | -0·104 (-0·224 to 0·016) | 0·09 | -0·11 | -0·189 (-0·310 to -0·068) | **0·002** | -0·20 |  |

Bold p-value = significant (<0·05) difference between conditions.

Abbreviations: B, Beta; BMIz, body mass index z-score; CI, confidence interval; ES, effect size; HPSF, Healthy Primary School of the Future;.
