# Supplementary material for: Battling the obesity epidemic with a school-based intervention: Long-term effects of a quasi-experimental study
Source: PLoS One. 2022 Sep 27;17(9):e0272291. doi: 10.1371/journal.pone.0272291 (PMC9514666; doi:10.1371/journal.pone.0272291)
Supplement: S5 Appendix — (DOCX) [file pone.0272291.s005.docx]

**S5. Estimated intervention effects after one, two, three and four years exposure**

**Table S5a: Estimated intervention effects after one, two, three and four years exposure on PA behaviours and dietary behaviours**

|  | | **Full HPSF vs control** | | | **Partial HPSF vs control** | | | |  |
| --- | --- | --- | --- | --- | --- | --- | --- | --- | --- |
|  |  | **B (95% CI)** | ***p*** | **ES** | **B (95% CI)** | ***p*** | **ES** |  |  |
| **Physical activity** | | | | | | | | | |
| **Sedentary behaviour**  **(n=1725)** | **E1** | -0·475 (-1·477 to 0·527) | 0·35 | -0·08 | 0·206 (-0·808 to 1·219) | 0·69 | 0·04 |  |  |
|  | **E2** | -0·873 (-1·948 to 0·203) | 0·11 | -0·15 | -0·085 (-1·155 to 0·986) | 0·88 | -0·02 |  |  |
|  | **E3** | 0·158 (-1·602 to 1·918) | 0·86 | 0·03 | 0·631 (-1·300 to 2·562) | 0·52 | 0·11 |  |  |
|  | **E4** | 0·606 (-0·750 to 1·962) | 0·38 | 0·11 | 1·422 (0·156 to 2·687) | **0·028** | 0·25 |  |  |
| **LPA**  **(n=1725)** | **E1** | 0·260 (-0·533 to 1·052) | 0·52 | 0·06 | -0·197 (-0·998 to 0·605) | 0·63 | -0·05 |  |  |
|  | **E2** | 0·801 (-0·036 to 1·639) | 0·061 | 0·18 | -0·060 (-0·891 to 0·772) | 0·89 | -0·01 |  |  |
|  | **E3** | 0·486 (-0·867 to 1·840) | 0·48 | 0·11 | -0·254 (-1·741 to 1·233) | 0·79 | -0·06 |  |  |
|  | **E4** | -0·620 (-1·657 to 0·417) | 0·24 | -0·14 | -1·124 (-2·090 to -0·157) | **0·023** | -0·26 |  |  |
| **MVPA**  **(n=1725)** | **E1** | 0·212 (-0·203 to 0·628) | 0·32 | 0·09 | 0·007 (-0·415 to 0·429) | 0·97 | 0·00 |  |  |
|  | **E2** | 0·055 (-0·390 to 0·500) | 0·81 | 0·02 | 0·162 (-0·282 to 0·607) | 0·48 | 0·07 |  |  |
|  | **E3** | -0·581 (-1·294 to 0·133) | 0·11 | -0·24 | -0·269 (-1·053 to 0·516) | 0·50 | -0·11 |  |  |
|  | **E4** | 0·004 (-0·554 to 0·563) | 0·99 | 0·00 | -0·270 (-0·793 to 0·252) | 0·31 | -0·11 |  |  |
| **Dietary behaviours** | | | | | | | | | |
| **School water consumption**  **(n=1840)** | **E1** | 0·795 (0·609 to 0·981) | **0·000** | 0·68 |  | | |  |  |
|  | **E2** | 0·811 (0·605 to 1·017) | **0·000** | 0·69 |  |  |  |  |  |
|  | **E3** | 0·572 (0·360 to 0·783) | **0·000** | 0·49 |  |  |  |  |  |
|  | **E4** | 0·540 (0·334 to 0·747) | **0·000** | 0·46 |  |  |  |  |  |
| **Healthy dietary behaviour**  **(n=1527)** | **E1** | 0·177 (0·032 to 0·323) | **0·017** | 0·18 |  |  |  |  |  |
|  | **E2** | 0·089 (-0·065 to 0·244) | 0·26 | 0·09 |  |  |  |  |  |
|  | **E3** | -0·042 (-0·224 to 0·140) | 0·65 | -0·04 |  |  |  |  |  |
|  | **E4** | -0·051 (-0·261 to 0·159) | 0·63 | -0·05 |  |  |  |  |  |
| **Unhealthy dietary behaviour**  **(n=1516)** | **E1** | -0·116 (-0·229 to -0·003) | **0·044** | -0·18 |  |  |  |  |  |
|  | **E2** | -0·117 (-0·238 to 0·003) | 0·056 | -0·18 |  |  |  |  |  |
|  | **E3** | -0·184 (-0·327 to -0·041) | **0·012** | -0·28 |  |  |  |  |  |
|  | **E4** | -0·049 (-0·215 to 0·116) | 0·56 | -0·07 |  |  |  |  |  |

Bold p-value = significant (≤0·05) difference between conditions.

Abbreviations: B, Beta; BMI, body mass index; CI, confidence interval; ES, effect size; HPSF, Healthy Primary School of the Future; LPA, light physical activity; MVPA, moderate to vigorous physical activity.

**Table S5b: Estimated intervention effects after one, two, three and four years exposure on lunch intake**

|  |  | **Full HPSF vs control** | |
| --- | --- | --- | --- |
|  |  | **OR (95% CI)** | ***p*** |
| **Fruit (% yes)**  **(n=2032)** | **E1** | 1·740 (1·191 to 2·541) | **0·004** |
|  | **E2** | 0·890 (0·611 to 1·298) | 0·546 |
|  | **E3** | 0·726 (0·482 to 1·093) | 0·125 |
|  | **E4** | 0·432 (0·267 to 0·698) | **0·001** |
| **Vegetables (% yes)**  **(n=2034)** | **E1** | 2·575 (1·707 to 3·888) | **0·000** |
|  | **E2** | 3·251 (2·130 to 4·963) | **0·000** |
|  | **E3** | 1·782 (1·151 to 2·759) | **0·010** |
|  | **E4** | 1·605 (0·970 to 2·656) | 0·065 |
| **Grains (% yes)**  **(n=2033)** | **E1** | 0·535 (0·291 to 0·983) | **0·044** |
|  | **E2** | 0·629 (0·342 to 1·160) | 0·138 |
|  | **E3** | 0·876 (0·459 to 1·674) | 0·690 |
|  | **E4** | 0·921 (0·433 to 1·962) | 0·832 |
| **Dairy (% yes)**  **(n=2033)** | **E1** | 3·040 (2·074 to 4·461) | **0·000** |
|  | **E2** | 2·581 (1·761 to 3·778) | **0·000** |
|  | **E3** | 1·904 (1·269 to 2·854) | **0·002** |
|  | **E4** | 1·647 (1·031 to 2·632) | **0·037** |
| **Water (% yes)**  **(n=2031)** | **E1** | 1·029 (0·705 to 1·503) | 0·882 |
|  | **E2** | 1·212 (0·824 to 1·781) | 0·329 |
|  | **E3** | 1·442 (0·961 to 2·163) | 0·077 |
|  | **E4** | 1·132 (0·715 to 1·790) | 0·597 |
| **Butter (% yes)**  **(n=2027)** | **E1** | 0·250 (0·174 to 0·359) | **0·000** |
|  | **E2** | 0·273 (0·188 to 0·395) | **0·000** |
|  | **E3** | 0·302 (0·202 to 0·451) | **0·000** |
|  | **E4** | 0·283 (0·176 to 0·454) | **0·000** |
| **Minimum two food groups at lunch (% yes)**  **(n=2037)** | **E1** | 3·037 (1·644 to 5·611) | **0·000** |
|  | **E2** | 2·956 (1·647 to 5·313) | **0·000** |
|  | **E3** | 2·312 (1·300 to 4·115) | **0·004** |
|  | **E4** | 1·289 (0·654 to 2·543) | 0·463 |

Bold p-value = significant (≤0·05) difference between conditions.

Abbreviations: CI, confidence interval; OR, odds ratio; HPSF, Healthy Primary School of the Future.
