# Supplementary material for: Battling the obesity epidemic with a school-based intervention: Long-term effects of a quasi-experimental study
Source: PLoS One. 2022 Sep 27;17(9):e0272291. doi: 10.1371/journal.pone.0272291 (PMC9514666; doi:10.1371/journal.pone.0272291)
Supplement: Attachment — Submitted filename: Rebuttal letter Plos Medicine_Plos One_Def.pdf [file pone.0272291.s006.pdf]

Maastricht, September 2021

Dear Dr. Jay-ar Formentera Medes,

We thank you for considering our manuscript entitled “Battling the obesity epidemic with a school-based intervention: Long-term effects of a quasi-experimental study” for consideration to Plos One, and for giving us the opportunity to react to the issues raised by the reviewers. We thank the reviewers for their extensive comments on our manuscript. Based on the comments, we were able to further improve our manuscript.

All changes made when revising the manuscript have been highlighted in the revised manuscript. In our response to the reviewers, we provide a point-by-point response to the reviewers' comments.

We believe we have addressed the comments satisfactorily, and hope that the article is suitable for publication in Plos One.

Yours sincerely, on behalf of the co-authors,

Dr. Maartje Willeboordse, Corresponding author

Reviewer 1

1. On Page 7, it is stated that data was gathered annually. However, some of the analyses (e.g. in Figures 3 & 4) display only figures for Years 0, 1 and 4. Is there any reason why the full range of data was not given?

Reply: The analyses over all years was only presented for anthropometric outcome measures, and not for dietary and PA behavior related outcome measures, as we followed our initial hypothesis in reporting data, which stated that improvements in dietary and PA behaviours will improve after one year of exposure, and remain sustained afterwards. For anthropometric measures on the other hand, we hypothesise increasing intervention effects on BMIz and WC with increasing intervention exposure. By doing this, we also believe that we prevent presenting tables with too much information.

2. On Page 7, it is stated that "...all analyses are based on an intention-to-treat principle; participants are included if they completed at least one measurement"; it might be clarified as to what "measurement" refers to here - for all of anthropometry, accelerometry & questionnaires covariates? The proportion of missing (imputed) covariates might also be stated, for each category. A couple of figures for questionnaire responses were provided on Page 17, but this might be done more fully and systematically.

Reply: In our intention-to-treat analysis we included all participants who were initially assigned to the intervention condition and gave informed consent. As missing data was imputed, participants had to engage in minimally one annual measurement round, as information on minimally one covariate and/or outcome variables was needed to impute the other missing values. This was adjusted on page 7, line 207.

As our study has an open cohort design, inclusion of participants was continuous, meaning that also children who entered school during the course of the study could enroll. Children who graduated or left school during the study period were not followed up. Following this design, data was not collected for all participants annually. As this is an uncommon design in scientific research, we chose to display average annual missing data for each type of measurement on page 9 line 244-246, as this best reflects the true amount of missing data.

3. Is there a reason why a two-level linear mixed model analysis was chosen, instead of a three-level one with the school as the highest level? This might be commented on.

Reply: After numerous discussions with our statistician, we found a two level linear mixed model to be the best fit for our dataset. With only eight included schools in our study, this amount was too small to adjust for in a three level design. Intervention condition was included as a fixed factor in our model, which is also determined at school level.

4. It is stated that weather variables were also considered for analyses regarding PA behaviours. It might be briefly commented as to whether the schools have indoor facilities allowing exercise (e.g. gyms, halls, other covered areas), and whether students would be allowed access during lunch breaks in the event of inclement weather.

Reply: Schools indeed have indoor facilities allowing exercise and play. On rainy days schools facilitated children to play and exercise indoors. This was included in our manuscript in page 7 line 189.

5. The difference in baseline BMIz between the full HPSF cohort (0.04 mean), and the partial/control cohorts (0.12/0.21 means) appears almost significant (Figure 2). From what could be understood, this initial baseline difference in BMIz would imply that a marginal improvement in BMIz - for the full HPSF cohort - would have resulted in a significant difference over the years, i.e. the effectiveness of the intervention for the BMIz outcome is advantaged by differences in initial BMIz. Moreover, it is stated that baseline BMIz is considered as a

covariate only for behavioural outcomes (Page 7). The omission of baseline BMIz in analyzing the BMIz outcome might thus be explained further, over and above the brief mention as a limitation due to lack of randomization.

Reply: There is indeed a baseline difference in BMIz between groups, which could have been caused by the lack of randomization. Nevertheless, in all type of analyses we conducted, an automatic correction for baseline score is included, which is equivalent to the use of change from baseline score. This is added in the method section in page 7, line 216. For this reason, we chose to not include baseline BMIz as a covariate in the analyses with BMIz as a primary outcome, as this would mean a double correction for BMIz.

6. For the secondary analyses, it might be considered to present the trends in the same format as Figure 2.

Reply: We chose to display primary and secondary outcome measures differently following our initial hypothesis. We hypothesized that improvements in the secondary outcome variables (dietary and PA behaviours) will improve after one year of exposure, and remain sustained afterwards. For the (primary) anthropometric measures on the other hand, we hypothesized increasing intervention effects on BMIz and WC with increasing intervention exposure. Therefore, we chose different analyses techniques for anthropometric variables and secondary outcome variables. Anthropometric outcomes were analysed as trend analyses, whereas secondary outcome variables where analysed as regular repeated measurement and shown after one and four years following our initial hypothesis.

7. For Figure 4, is there any particular reason why the data is presented as bar charts instead of the line graphs (with confidence intervals) as used for Figures 2 & 3? It would appear that the requisite confidence intervals have been computed, to determine significance; as such, they might be displayed even if the bar chart format is retained.

Reply: In figure 4 we present the raw data of binary outcome measures, therefore a bar chart is more suitable than a line graph. For raw data of binary outcome measures (e.g. eating fruit during lunch: yes or no) it is not possible to show confidence intervals. The confidence intervals over the Odds Ratios, which were used to calculate significance, are shown in table 4.

8. In Table S2, it might be clarified as to what the "school groups" listed in the table cells refer to. Given that the range appears to be from 1 to 8, do these numbers refer to the eight schools (2 full intervention, 2 partial, 4 control)? If so, the correspondence between each school (group) index and intervention group might be explicitly stated. Furthermore, might the school (group) ranges then imply that certain types of measurements were unavailable for entire schools (e.g. the "4-8" under the child questionnaire column, means that child questionnaires were never given to subjects from school [group] 1-3), and that these missing values were all imputed for statistical modelling purposes? This might be clarified.

Reply: We agree with the reviewer that this was not clear from Table S2. All measurements were conducted equally in all 8 included schools. Children in Dutch schools are divided over 8 groups, whereby children in group 1 and 2 are aged 4-6 years old, which correspond to kindergarten, and children in Dutch groups 3-8 are aged 6-12 years which corresponds to the International recognized grade 1-6. We adjusted this at page 28, line 15-17.

Minor issues

9. On Page 17, there appears to be additional brackets after "...and BMIz at E0 (only for the behavioural outcomes)".

Reply: Thank you for notifying this inconsistency, we adjusted this at page 17, line 423.

Reviewer 2

1. Ethics approval was waived. Was this because these measurements were done as part of the school day or by school staff? Please provide more information on this because researchers took measurements and parental consent was obtained. Also Talk about the consent process? Who did this and how did the parents get the information (was it via teachers, the school or from the research team)? Was it part of the agreement when a pupil entered one of the eight schools that they would have these measurements taken? The actual 'doing' on this when the ethics was waived would be very beneficial to other researchers.

Reply: In the Netherlands, The Medical Ethical Committee can waive ethical approval if studies are not part of the Medical Research Involving Human Subjects Act (see also: https://english.ccmo.nl/investigators/legal-framework-for-medical-scientific-research/your-research-is-it-subject-to-the-wmo-or-not). This study was not part of this Act, as it did not fall under medical scientific research, and participants were not subject to procedures or required to follow rules of behavior. The measurements were done by researchers and on a voluntary basis, but the interventions were part of the school day for all children (regardless of informed consent for scientific research) and executed by school staff and some external partners (e.g. pedagogical employees, sport partners and logistic partners). In page 4, line 137-138 it is explained that parents or guardians had to sign an informed consent. Children were informed about the study, yet only needed to sign an informed consent if they were aged 12 years or older at the start. In reference 10 the recruitment process was explained in detail. We do not describe this elaborate recruitment procedure in the current manuscript to prevent overlap with the other published article, and to limit the word count.

2. Was child assent taken?

Reply: Children were informed about the study, yet only needed to sign an informed consent if they were aged 12 years or older at the start. This was only the case for 5 children in our study, as the age range of children in Dutch primary schools at the beginning of the semester ranges usually between 4 and 11 years. The recruitment procedure including assent is described in reference 10 of Willeboordse et al.

3. Was a sensitivity analysis done for those that were exposed to one year versus 4 years of the intervention?

Reply: We performed a sensitivity analysis with children who were exposed for the full four years. This sensitivity analysis resulted in comparable effect sizes in both full and partial HPSFs. This sensitivity analysis is described in the method sections at page 8, line 226-228, and in the result section at page 10 line 271-273. We deemed it unnecessary to perform a sensitivity analyses for children who were exposed for only one year, as the amount of children in this type of analysis hardly differs from the amount of children in the primary analysis.

4. This may e a point in one of the supporting papers but was any advice given to control schools about not taking on other healthy lifestyles programmes over the 4 years? What benefits did the control school get?

Reply: Control schools maintained the school curriculum that is currently common practice in the Netherlands. Following this regular school curriculum, they could take on other healthy lifestyle programs if they deemed this necessary for their school, thereby reflecting a real-life school situation, which was the aim of our quasi-experimental design. This was described in reference 10, but also newly added in our manuscript at page 6, line 178-179. The control schools didn't receive any benefit from participating in this study.

5. There are a lot of abbreviations in the results section that make some of the paragraphs hard to follow. e.g. the secondary analysis section.

Reply: We carefully checked the frequency of abbreviation in our entire manuscript. We only included abbreviations if they were used minimally 10 times in the manuscript. We deleted the abbreviation SES (Socioeconomic status) in the current version.

6. There are a lot of results presented in the tables in terms of secondary analysis. Could some of these be added to a Suppl document instead and/or chop up the tables into smaller more manageable tables and consider a different lay out to the tables that aligns more with tables from clinical trials.

Reply: We agree with the reviewer that our study contains many secondary outcome variables. The majority of the secondary outcome measures we which to measure, e.g. physical activity and nutrition behaviors, cannot be measured with one single outcome. Therefore, we chose to include a set of relevant outcome measures on these behaviours, to increase the reliability and validity of our study. Nevertheless, if the editor deems it necessary, we will ofcourse move some of our secondary outcome measures to the supplementary files.

Some of the tables have indeed a slightly uncommon lay out, which might be caused by the uncommon open cohort design of our study, in which we work with exposure to the intervention, and not the chronological time variable. The baseline exposure (E0) is defined by the first moment children were exposed to the school environment starting from September 2015, which is not necessarily equal to the first measurement children attended. If the reviewer has specific advice to improve the layout of our tables, we highly welcome this.

Reviewer 3

Major points

1. Were baseline outcomes adjusted for in relevant analyses? For example, was baseline BMIz controlled for in the analysis of BMIz as the primary outcome at the follow up? This is important because as the authors said 'At baseline, children in control schools showed less favourable dietary and PA behaviours, were more often overweight and obese, and had a lower SES compared with the full and partial HPSFs'. Table 2 shows that the mean baseline BMIz in the full HPSF was 0.04 but 0.12 and 0.21 in Partial HPSF and Control respectively (largely unbalanced). If this is only a reporting issue, please clarify this in the Methods section.

Reply: There is indeed a baseline difference in BMIz between groups, which could have been caused by the lack of randomization. Nevertheless, in all type of analyses we conducted, an automatic correction for baseline score is included, which is equivalent to the use of change from baseline score. This is added in the method section in page 7, line 216.

2. There should have been a clear distinction between long term intervention and long term follow up. If the intervention wasn't stopped at the end of year 1, we can't say the follow up was 1/2/3/4 years in length. The Introduction didn't make it clear as to which research gap the study was aimed to address (intervention length or the sustainability of the intervention effect?) Based on the current Introduction, the research team seemed to evaluate long term effect of a school based intervention (so focused on the sustainability of intervention effect) as they argued 'school based interventions can only be of societal and clinical relevance if intervention effects are maintained for a prolonged time'. I would expect follow up measures to be undertaken for example, in 1, 2, 3, 4…years following the completion of the intervention to assess the impact sustainability. However, I also noted that in S1, the authors said 'The majority of studies have an intervention duration ≤24 months, or rely on small study populations (figure S1); therefore, robust evidence on the trend in intervention effects with continued intervention implementation is lacking'. So please make it clear in the Introduction what methodological/research gaps this study was aimed to address and why. I like the statement given in the Discussion which I think correctly described the study aim 'The results…described increasing intervention effects on BMIz and WC with increasing intervention exposure'.

Reply: In our study, we aim to measure the effects of long-term exposure to the intervention on anthropometry and dietary- and physical activity behaviours. Our design does not allow studying the sustainability of the intervention effects after discontinuation of the intervention. We agree with the reviewer that this was not clearly formulated in all sections of the introduction section; we made adjustments in several sections in the introduction (p3, line 101-102, 107 and 114). We thank the reviewer for the compliments on our discussion section.

Minor revisions
Abstract

3. Primary and secondary outcomes were not defined in the Abstract.

Reply: We thank the reviewer for noticing this inaccuracy; we added this in the abstract at page 2, line 58-59

4. What do you mean by 'average exposure to the school condition'?

Reply: The average exposure to the school condition averages the amount of years children were exposed to the school condition (e.g. control, full HPSF or partial HPSF). As our study has an open cohort design, inclusion of participants was continuous, meaning that also children who entered school during the course of the study could enroll. Children who graduated or left school during the study period were not followed up. Therefore, not all participants were exposed to the school

condition for the full study duration. The amount of time children were exposed to HPSF varies from 0-4 years and is expressed by the variable exposure (E0 to E4). The baseline exposure (E0) is defined by the first moment children were exposed to the school environment starting from September 2015, which is not necessarily equal to the first measurement children attended. This is explained in the method section at page 4, line 128-135.

5. Please correct this sentence 'effective in bringing unfavourable changes in …'. Did you mean to say 'favourable'?

Reply: In figure 2 it is visible that the intervention did not actively decrease BMIz score in children, but children in control group continuously increased their BMIz score. In other words, the intervention condition prevented an unfavorable increase in BMIz, which was clearly visible in the control group. Therefore, we think that the phrase 'This school-based health-promoting intervention is effective in bringing unfavourable changes in body composition to a halt in both the short and long term', reflects the findings of our study correctly.

6. I am not sure about the term 'robust evidence' as a quasi-experiment but the strength of using a quasi-experiment (especially higher ecological validity) can be discussed in the main paper.

Reply: We thank the reviewer for pointing out this strength of our study design, and included this in our discussion at page 17, line 418.

Introduction

7. Reference no.5 was published 8 years ago and the evidence was specifically focused on educational interventions only. I would suggest replacing this reference with a more recent review such as the 2019 Cochrane review.

Reply: We agree with the reviewer that this is not an optimal reference for this section. We deleted reference 5 and included the 2019 Cochrane review of Brown et al.

8. Reference is missing for this statement: School-based interventions can only be of societal and clinical relevance if intervention effects are maintained for a prolonged time.

Reply: We added a reference to this statement and slightly adjusted the sentence (page 3, line 101-102).

Methods

9. Although details of the study design and intervention have been published elsewhere, some brief description in this paper would be helpful, so readers don't need to search and read other papers in order to fully understand this paper. For example, what components were included in HPSF, who delivered the intervention and intervention length etc.

Reply: We agree with the reviewer that some more information on the study design and intervention could be added to the current manuscript. We included study length and intervention delivery in the method section (p. 6 line 182, page 7 line 197). The intervention components are described in table 1.

10. Please specify if the 'trained researchers' were blinded to allocation?

Reply: Trained researchers were not blinded to allocation, as this was not possible in this type of research. This was added in page 7, line 197.

11. Including 'age in years', instead of age in months might be a potential limitation to be discussed for this age group.

Reply: As the age range of our study ranged between 4-13 years, we considered 'age in years' to be specific enough.

12. What indicator(s) of SES was used?

Reply: In supplement S2 (page 29, line 53-58) we explain that socioeconomic status was calculated using a standardized score of maternal education level, paternal education level and household income adjusted for household size.[43] Mean scores were categorized into low, middle and high, based on tertiles.

13. Whether/how school clustering was addressed in the analysis? If not, why?

Reply: After numerous discussions with our statistician, we found a two level linear mixed model to be the best fit for our dataset. With only eight included schools in our study, this amount was too small to adjust for in a three level design. Intervention condition was already included as a fixed factor in our model, which is also determined at school level.

Results

14. In the main text, results were not reported consistently. In some places, CIs and P values were specified alongside effect sizes but in other places, only effect sizes were given.

Reply: We agree with reviewer 3 that the presentation of results was not consequent. In the current version of our manuscript, full specification of analyses are given for the primary outcome measure in the text (e.g. Beta, confidence intervals, effect sizes and p-value). For all secondary analyses and the stratification analysis, full specification of analyses is given in tables, and a lower amount of specification is shown in the text (e.g. effect size and p-value in case of significant findings) to ensure that the text remains easy to read. The sensitivity analyses are not shown separately in tables, therefore, full specification of these analyses are also given in the text.

15. For all results tables, please indicate case number for each analytical group. For example for Table 3, what was 'n' referred to? How many cases were in the Full HPSF and Control for each row of analysis?

Reply: The n in all tables correspondents to the total amount of unique participants included in the specific analyses. As we used multiple imputations if data was missing, this amount of participant does not vary much between analyses. This advice of reviewer 3 is not in in line with the advice of reviewer 2, who advised us to limit the amount of information in the tables in the result section. Therefore, we chose to not add this information to the tables.

Discussion

16. Can the authors comment on the cost (or cost effectiveness) on this 4-year intervention? Effect size and value for money are both important considerations for decision makers.

Reply: We agree with the reviewer that both effect size and value for money are important considerations in policy making. Therefore, we included in the discussion on page 17 line 401-408 information on the short- and long-term cost-effectiveness of this intervention. In the related references of Oosterhoff et al, all details about the costs and cost-effectiveness calculation can be found.
