# Supplementary material for: Battling the obesity epidemic with a school-based intervention: Long-term effects of a quasi-experimental study
Source: PLoS One. 2022 Sep 27;17(9):e0272291. doi: 10.1371/journal.pone.0272291 (PMC9514666; doi:10.1371/journal.pone.0272291)
Supplement: Attachment — Submitted filename: Plos One Review comments 20220119.docx [file pone.0272291.s007.docx]

**Review Comments**

PONE-D-21-31405 Battling the obesity epidemic with a school-based intervention: Long-term effects of a quasi-experimental study

**Major comments:**

1. Power calculations justifying sample size are not presented, as well as justification for the effect size included in the calculation. Have they been published elsewhere? A brief description in this paper would be helpful.
2. The rate of attrition (drop out) could be clarified. What was the rate of attrition by the study arms? What was the attrition rate assumed for power calculations? Is attrition randomly distributed? If there is non-random attrition, what statistical techniques are used to identify and adjust for the attrition bias?
3. Any comments on spill-overs? How is the contamination controlled for (e.g. the participation of three comparison schools with the European School Fruit and Vegetables Scheme)?
4. For a combined table on baseline characteristics, the last column usually gives the p value for the comparison between study groups. Why is the p value on baseline differences not presented and only the mean difference is reported?
5. Can the authors report both adjusted and unadjusted models, to avoid reporting bias? Do both adjusted and unadjusted analyses yield the same results?
6. “Comparison schools” instead of “control schools” as this is not an RCT?
7. Can the authors comment on food waste in this 4-year intervention? Food waste in school nutrition programs has received increasing attention.

**Minor comments:**

**Introduction**

8. Reference is missing for this statement: Current evidence suggests that school-based interventions show small favourable effects in terms of body mass index (BMI), and dietary and physical activity (PA) behaviours.
9. For L109-L113, is there any particular reason why result on BMIz in the short term evaluation is not reported? It would appear that effects on BMIz only started to show after two years, while effects on BMIz was significant from E1 to E4 as reported in the Results section. This could be clarified.
10. The statement “We hypothesise that improvements in dietary and PA behaviours will improve after one year of exposure…” (L117) could be clarified. Does it refer to an increasing intervention effect?

**Study design**

11. The numbers reported in L153-L157 are not lined up with numbers on E0-E4 in *Participation expressed in exposure (in years)* (Figure 1). This could be clarified.

**Results**

12. Was the stratification by gender specified in the pre-analysis plan? (L269-L270)
13. Results on PA behaviors at E4 appears to be counter-intuitive (L294-L295). This could be commented on in the Discussion section.
14. How are Figure 3 & Figure 4 referenced in the text?

**Discussion**

15. On L390, “water and dairy consumption instead of soft drinks” is stated. Is a decrease in soft drinks detected to support the statement? This could be reported as in the current study, the greatest effect was found in children’s water consumption.
