# Supplementary material for: Battling the obesity epidemic with a school-based intervention: Long-term effects of a quasi-experimental study
Source: PLoS One. 2022 Sep 27;17(9):e0272291. doi: 10.1371/journal.pone.0272291 (PMC9514666; doi:10.1371/journal.pone.0272291)
Supplement: Attachment — Submitted filename: Response to reviewers.docx [file pone.0272291.s008.docx]

**Review Comments**

PONE-D-21-31405 Battling the obesity epidemic with a school-based intervention: Long-term effects of a quasi-experimental study

**Major comments:**

1. *Power calculations justifying sample size are not presented, as well as justification for the effect size included in the calculation. Have they been published elsewhere? A brief description in this paper would be helpful.*

Response: The power calculation has been published in our original design paper (reference 11, Willeboordse et al.). We added a reference to the original power calculation in the method section of the current article (page 4, line 124).

2. *The rate of attrition (drop out) could be clarified. What was the rate of attrition by the study arms? What was the attrition rate assumed for power calculations? Is attrition randomly distributed? If there is non-random attrition, what statistical techniques are used to identify and adjust for the attrition bias?*

Response: As our study follows a dynamic open cohort design in which we use the variable “exposure to the intervention in years” instead of the chronological variable “time of measurement”, reporting about attrition is slightly different from the reporting in studies with a closed cohort design. At page 6 (lines 160-168), we describe the amount of attrition specified per study arm, including reasons for attrition. We added the relative percentages of attrition to this section, so it is easier to assess if the amount of attrition and reasons for attrition differed between study arms.

We used multiple imputation for baseline data and a likelihood-based approach for missing outcome data. These methods assume that the missing values are at random (MAR), which means they are allowed to depend on observed variables, but not on unobserved variables. Additionally, we performed a sensitivity analysis for the primary outcome variable in which we only included children who were exposed for the full four years. With this analysis, we could assess whether attrition affected our study results. This sensitivity analysis resulted in comparable effect sizes in both full and partial HPSFs. This sensitivity analysis is described in the method sections at page 8, lines 232-234, and in the result section at page 10 lines 277-279.

As for the amount of missingness, this exceeded the dropout rate of 20% we anticipated in the sample size calculation. We accounted partially for this by using multiple imputation and a mixed model using a likelihood-based approach for missing outcome values. This resulted in an intention-to-treat analysis, where participants with at least one outcome measurement were included. Additionally, we originally planned to include at least 100 participants per school. In the end, we included many more participants per school, thereby correcting for the higher amount of loss due to attrition. This discussion point was added to the manuscript at page 17, lines 418-421.

3. *Any comments on spill-overs? How is the contamination controlled for (e.g. the participation of three comparison schools with the European School Fruit and Vegetables Scheme)?*

Response: In this quasi-experimental trial, we aimed to compare the effects of the intervention with the real-life situation of school health promotion in the Netherlands. In the Dutch setting, schools are free to decide if they implement health-promoting interventions. Therefore, the researchers did not prohibit control schools to engage in health-promoting interventions. This feature of our research design is discussed in our original design paper (reference 11, Willeboordse et al.).

During the four-year period, the control schools maintained their regular school curriculum and school hours. Children continued to consume their lunch brought from home and engaged in free play supervised by volunteers, as is common in Dutch schools. This is described in the methods of the current article at page 4, lines 140-143. Control schools implemented some health-promoting interventions in line with other schools in the Netherlands. This was monitored closely by the research time by means of an annual questionnaire for school directors. As the degree to which control schools implemented health-promoting interventions in the Netherlands did not differ much between schools and reflected the degree of implementation of other schools in the region, we decided not to control for this in the analysis. We describe in the discussion section of the current article that the participation of three control schools in the European School Fruit and Vegetables Scheme could have affected the results on healthy dietary behaviours (page 16, lines 380-382).

4. *For a combined table on baseline characteristics, the last column usually gives the p value for the comparison between study groups. Why is the p value on baseline differences not presented and only the mean difference is reported?*

Response: We have chosen not to report the p-values, because small, non-clinically relevant differences will also be statistically significant due to the large group sizes. Instead of statistical significance, we focused on the clinical relevance of the differences and reported those that were, to our opinion, meaningful in practice. As all baseline characteristics are shown in Table 2, readers might check the differences themselves. In addition, in all our analyses, we chose to control for the baseline values of the outcomes, and to include covariates based on their possible correlation with variable and outcomes, regardless of whether the baseline data exhibit imbalances in statistical tests. Therefore, even if there is an imbalance in some characteristics at baseline, this would probably have affected the outcomes at baseline, for which we corrected in our analysis. This is also discussed in the discussion section (page 17, lines 430-434).

5. *Can the authors report both adjusted and unadjusted models, to avoid reporting bias? Do both adjusted and unadjusted analyses yield the same results?*

Response: Unadjusted models were not reported, as this is not a randomised study. Due to baseline imbalance, the unadjusted effects are most likely biased due to confounding. The models we used also corrected for differences in the outcome at baseline, which is recommended for non-randomised (less bias) as well as for randomised studies (more power due to the correlation between outcome at baseline and after treatment).

6. *“Comparison schools” instead of “control schools” as this is not an RCT?*

Response: We agree with the reviewer that comparison schools is a more suitable terminology for a non-randomised study than control schools. However, in all other published articles of this trial we use the terminology “control schools”. As we aim to align the terminology and results of this study with other published articles, we prefer to maintain the term control schools.

7. *Can the authors comment on food waste in this 4-year intervention? Food waste in school nutrition programs has received increasing attention.*

Response: We agree with the reviewer that it is of high importance to minimalise food waste in school food nutrition programs. In the Healthy Primary School of the Future, different strategies were implemented to minimalise food waste. It goes beyond the scope of the current article to include these strategies in the manuscript. We added food waste to the list of societal benefits and revenues that should be considered when implementing school-based health-promoting interventions (page 17, lines 406).

**Minor comments:**

**Introduction**

8. *Reference is missing for this statement: Current evidence suggests that school-based interventions show small favourable effects in terms of body mass index (BMI), and dietary and physical activity (PA) behaviours.*

Response: We added references for this statement at page 3, line 91.

9. *For L109-L113, is there any particular reason why result on BMIz in the short term evaluation is not reported? It would appear that effects on BMIz only started to show after two years, while effects on BMIz was significant from E1 to E4 as reported in the Results section. This could be clarified.*

Response: In the study of Bartelink et al, beneficial intervention effects of the intervention on BMIz were visible both one and two years after the start of implementation. This is adjusted in the manuscript on page 3, lines 109-113.

10. *The statement “We hypothesise that improvements in dietary and PA behaviours will improve after one year of exposure…” (L117) could be clarified. Does it refer to an increasing intervention effect?*

Response: We thank the reviewer for carefully reading this section. The formulation of this sentence was not correct, as we aimed to explain here that we do not expect increasing intervention effects for PA and dietary behaviours. We adjusted this sentence (page 3, lines 117-118), it now reads “We hypothesise that improvements in dietary and PA behaviours will be visible after one year of exposure, and remain sustained.”

**Study design**

11. *The numbers reported in L153-L157 are not lined up with numbers on E0-E4 in Participation expressed in exposure (in years) (Figure 1). This could be clarified.*

Response: In lines 153-157 we report the amount of children included at each chronological time point (i.e., T0, T1, T2, T3, and T4) separately for each exposure moment (i.e., E0, E1, E2, E3, and E4). The children which had to be excluded from analyses were not included in the numbers in lines 153-157, whilst in Figure 1 the excluded children were included. We added the amount of excluded children to the text at lines 157-159 so that the numbers between the text and figure line up (page 6). In addition, we found one error in the numbers reported in line 153. The amount of children with 0 years exposure enrolled during T4 was 25, instead of 30. This was adjusted in the manuscript (page 6, line 153).

A detailed calculation is given below:

The n=900 participants which were exposed for the full your years, coincides between Figure 1 and the text in lines 153-157.

The n=1392 participants which were exposed for three years (Figure 1), is composed by adding the n=1120 participants which enrolled during T0 with the n=269 participants enrolled during T1, and n=3 participants which had to be excluded during final analyses.

The n=1807 participants which were exposed for two years (Figure 1) is composed by adding the n=1320 participants enrolled during T0, n=287 participants enrolled during T1, n=196 participants enrolled during T2, and n=4 participants which had to be excluded during final analyses.

The n=2157 participants which were exposed for one year (Figure 1), is composed by adding the n=1553 enrolled during T0, n=309 enrolled during T1, n=200 enrolled during T2, n=90 enrolled during T3, and n=5 participants which had to be excluded during final analyses.

The n=2349 participants which were exposed for zero years (Figure 1), is composed by adding the n=1699 participants enrolled during T0, n=233 participants enrolled during T1, n=200 participants enrolled during T2, n=91 enrolled during T3, n=25 enrolled during T4, and n=101 participants which had to be excluded during final analyses.

The total amount of persons excluded during final analyses is 113 (i.e., n=101 with E0, n=5 with E1, n=4 with E2, n=3 with E1, and n=0 with E4), this aligns with the amount of children excluded shown in Figure 1 (i.e., n=38 in full HPSF, n=27 in partial HPSF, and n=48 in control schools, which adds up to 113).

**Results**

12. *Was the stratification by gender specified in the pre-analysis plan? (L269-L270)*

Response: Gender stratification was not included in our original analysis plan. However, as gender stratification of the primary outcome results is strongly recommended by the PLOS guidelines, we chose to include this in our analysis.

13. *Results on PA behaviours at E4 appears to be counter-intuitive (L294-L295). This could be commented on in the Discussion section.*

Response: We agree with the reviewer that results on PA behaviours in partial HPSFs are counter-intuitive. We added a comment on this in the discussion section (page 16, lines 384-386).

14. *How are Figure 3 & Figure 4 referenced in the text?*

Response: Figure 3 and 4 were referenced to on page 10, line 288. To increase visibility, we added new references to the figures on page 11, lines 294 and 303.

**Discussion**

15. *On L390, “water and dairy consumption instead of soft drinks” is stated. Is a decrease in soft drinks detected to support the statement? This could be reported as in the current study, the greatest effect was found in children’s water consumption.*

Response: We asked participants about water and milk consumption during school hours. We did not ask participants about sugar-sweetened beverages consumption during school hours. In the Netherlands, it is common for children to bring sugar-sweetened beverages to school, such as juice boxes and lemonade. Teachers at both full HPSFs reported a frequent daily consumption of sugar-sweetened beverages pre-intervention during school hours for a large proportion of children. This was also observed by the researchers during the T0 measurements. After implementation of the intervention, home-brought food products and beverages were no longer allowed at school, and children were solely offered water and milk during school hours. Consequently, water and dairy consumption during school hours increased. Although we cannot prove there was a decrease in sugar-sweetened beverages with quantitative data in this study, we deem it highly likely that sugar-sweetened beverages decreased during school hours. Therefore, we decided to leave this statement unchanged. We did change the wording soft drinks into sugar-sweetened beverages in page 17, line 399.
